# Tick Species Diversity and Molecular Identification of Spotted Fever Group Rickettsiae Collected from Migratory Birds Arriving from Africa

**DOI:** 10.3390/microorganisms11082036

**Published:** 2023-08-08

**Authors:** Elisa Mancuso, Marco Di Domenico, Luigina Di Gialleonardo, Michela Menegon, Luciano Toma, Marco Di Luca, Francesca Casale, Guido Di Donato, Laura D’Onofrio, Angela De Rosa, Sara Riello, Andrea Ferri, Lorenzo Serra, Federica Monaco

**Affiliations:** 1 Istituto Zooprofilattico Sperimentale dell’Abruzzo e del Molise “G. Caporale”, 64100 Teramo, Italy; m.didomenico@izs.it (M.D.D.); l.digialleonardo@izs.it (L.D.G.); gu.didonato@izs.it (G.D.D.); l.donofrio@izs.it (L.D.); a.derosa@izs.it (A.D.R.); f.monaco@izs.it (F.M.); 2 Dipartimento di Scienze Biomolecolari, Università di Urbino “Carlo Bo”, 61029 Urbino, Italy; 3 Dipartimento Malattie Infettive, Reparto Malattie Trasmesse da Vettori, Istituto Superiore di Sanità, 00161 Rome, Italy; michela.menegon@iss.it (M.M.); luciano.toma@iss.it (L.T.); marco.diluca@iss.it (M.D.L.); francesca.casale@guest.iss.it (F.C.); 4 Riserva Naturale Statale “Isole di Ventotene e Santo Stefano”, 04031 Ventotene, Italy; sarariello@gmail.com; 5 Area Avifauna Migratrice, Istituto Superiore per la Protezione e la Ricerca Ambientale (ISPRA), 40064 Ozzano dell’Emilia, Italy; andrea.ferri@isprambiente.it (A.F.); lorenzo.serra@isprambiente.it (L.S.)

**Keywords:** tick-borne pathogens, *Rickettsia*, migratory birds, zoonoses, Italy

## Abstract

The role of migratory birds in the spread of ticks and tick-borne pathogens along their routes from Africa to Europe is increasingly emerging. Wild birds can host several tick species, often infected by bacteria responsible for zoonoses. The aim of the study is to assess the possible introduction of exotic ticks carried by migratory birds into Italy from Africa and to detect the presence of *Rickettsia* species and *Coxiella burnetii* they may harbor. During a two-year survey, we collected ticks from migratory birds captured during their short stop-over on Ventotene Island. Specimens were first identified by morphology or sequencing molecular targets when needed, and then tested by real-time PCR for the presence of selected pathogens. A total of 91% of the collection consisted of sub-Saharan ticks, more than 50% of which were infected by *Rickettsia* species belonging to the spotted fever group, mainly represented by *R. aeschlimannii*. In contrast, the suspected *C. burnetii* detected in two soft ticks were confirmed as *Coxiella*-like endosymbionts and not the pathogen. Although there are still gaps in the knowledge of this dispersal process, our findings confirm the role of migratory birds in the spread of ticks and tick-borne pathogens, suggesting the need for a continuous surveillance to monitor the potential emergence of new diseases in Europe.

## 1. Introduction

Ticks are blood-feeding ectoparasites and major vectors of pathogens that cause infectious diseases worldwide in humans and animals, including mammals, birds, and reptiles, representing one of the most important vectors of infectious zoonotic diseases. There are about 900 different species of ticks having a wide geographical distribution. These parasites can be passively transported for long distances through the movement of their hosts, contributing to the dissemination of tick-borne pathogens. Birds are among the vertebrate animal hosts with the greatest mobility capabilities that allow them to overcome geographical barriers and spread parasites at different scales [1]. In particular, migratory birds cover regional and intercontinental distances twice a year in a short time during their migratory seasons. Indeed, they may leave their European breeding grounds during the Boreal winter season to reach the Mediterranean Basin (intra-Palearctic or short-distance migrants) or sub-Saharan areas (trans-Saharan or long-distance migrants) as their southernmost range, depending on their migratory strategy [2].

In the scenarios of global warming and habitats changing due to the pressure of human activities, ticks and tick-borne pathogens (TBPs) are expanding their geographical ranges implying, consequently, modifications to their ecological features, impacts on the dynamics of local host populations, and the emergence of human and livestock diseases [1]. Indeed, immatures of sub-Saharan Africa hard tick species belonging to *Hyalomma* and *Amblyomma* genera have been commonly found on migratory birds in Italy in the last decade [3,4,5,6,7,8], and a limited, but growing, number of adults have been reported on mammals around central and northern Europe [9,10,11,12]. These findings suggest the possibility of new introductions and the future spread of these two genera of ticks, very common in Africa, at higher latitudes due to new climatic and ecological conditions. The genus *Amblyomma* is one of the abundant and widespread hard tick genera (Ixodidae) with about 130 valid taxa [13] mainly distributed in the southern hemisphere. Some of the *Amblyomma* species are of medical and veterinary importance because they can serve as vectors and reservoirs of many pathogens as several *Rickettsia* and *Ehrlichia* species [14,15]. Similarly, the 27 species of *Hyalomma* ticks, widely distributed in Europe, Africa, and Asia, and well known to be competent vectors, such as of the Crimean Congo hemorrhagic fever virus (CCHFV) [16], are also vectors of various zoonotic bacteria [17,18,19]. These tick genera are usually two- and three-host ticks, with adults parasitizing large vertebrates and immatures searching for small hosts, including birds, which easily contribute to their spread over long distances.

Consequently, TBPs, such as those belonging to the genera *Rickettsia* and *Coxiella burnetii*, agents of widespread zoonotic diseases affecting humans and animals, can spread together with their vectors. Rickettsiae are small Gram-negative, obligate intracellular bacteria belonging to the order Rickettsiales, mainly transmitted by arthropod vectors. Members of this genus are classified, at present, into four groups: the spotted fever group (SFGR); typhus group (TGR); *R. bellii* group; and *R. canadensis* group [20]. The SFGR is the most abundant and widespread with 48 confirmed species that includes 17 predominant *Rickettsia* spp., which form five spatial clusters, each representing unique combinations of environmental and eco-climatic features [17]. Diseases caused by the SFGR are often difficult to detect since they are characterized by multiple non-specific symptoms, such as fever, headache, muscle pain, single or multiple eschars, regional swelling of lymph nodes, rash, or mild or moderately severe illness [21].

*C. burnetii* is an obligate intracellular bacterium belonging to the Legionellales order and an etiological agent of Q fever, a zoonotic disease distributed worldwide. Flu-like symptoms with possible complications concerning the lungs, liver, vascular system, and heart are the characteristic signs of the disease in humans. The role of ticks as vectors is still controversial because of the low-frequency detection in ticks and the difficulty in distinguishing between pathogenic *C. burnetii* and nonpathogenic *Coxiella*-like endosymbionts, which casts doubt on the relevance of ticks in the epidemiology of Q fever [22]. Emerging and re-emerging distributions and associated risks of these pathogens remains poorly understood [17,21]; hence, further studies are needed to investigate and prevent the consequences of the spread of new tick and pathogen species in naïve areas as an effect of global warming.

In our study, we collected ticks parasitizing wild birds during their migration from African non-breeding quarters to the breeding ones in Europe, in spring 2021 and 2022. The aim of this study is to provide an up-to-date overview of tick species and the related bacterial agents transmitted by ticks to migratory birds, particularly those wintering in sub-Saharan areas, with a focus on *C. burnetii* and *Rickettsia* species that can potentially be introduced from Africa to Europe.

## 2. Materials and Methods

**Figure 1:**
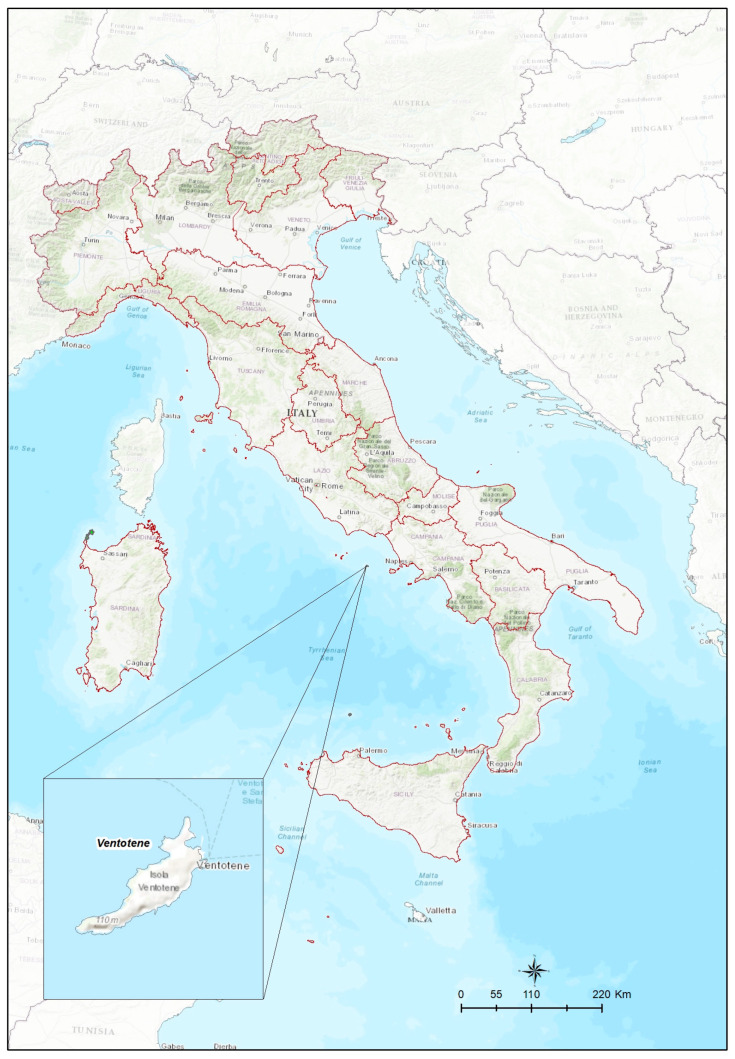
Localization of Ventotene Island: stop-over and tick-collection site during spring bird migration from Africa.

The collection of ticks from birds was conducted during the spring seasons 2021 and 2022 within the framework of bird ringing (bird banding) activities on Ventotene island (coordinates 40°47′11″ N, 13°24′55″ E) located in the central Tyrrhenian sea (Figure 1). The island is one of the most important stop-over sites in the Mediterranean for migratory birds, mainly Passeriformes, and hosts a ringing station operating since 1988 as part of a large-scale and long-term project coordinated by the Italian Institute for Environmental Protection and Research (ISPRA) to monitor the spring migration. The sampling period started the end of March and continued until the end of May during the seasonal peak of trans-Saharan migratory birds’ passage over the Island. It allowed us to focus on the collection of African ticks and TBPs transported from the endemic African areas. Birds were captured every day according to the weather conditions using nearly 350 m mist-net transects; they were handled for ringing procedures and checked for the presence of ticks. All the procedures were performed by authorized expert bird-ringers. Ticks were removed using a tick twister or tweezers and stored in Eppendorf tubes containing 70% ethanol at room temperature until processed. All the parasites collected from a single bird were stored in the same tube, recording the date and host species.

### Tick Identification and Pathogen Detection

Parasites were first classified according to life stage (larva, nymph, adult) and the morphologically was identified by using dichotomous published keys [23,24]. Ticks infesting migratory birds are mostly immature and their morphological identification at species level is often inconclusive. Therefore, they were individually subjected to nucleic acid extraction for molecular identification and screening for pathogen detection. Once taken out of the 70% ethanol, each tick was air-dried and homogenized in RLT lysis buffer provided by the BioSprint 96 One-For-All Vet kit (INDICAL BIOSCIENCE, Leipzig, Germany), using a 5 mm steel bead and Tissue-lyser LT (Qiagen, Hilden, Germany). The BS96 Vet 100 protocol for the extraction from animal tissues was applied according to the manufacturer’s instructions.

The molecular identification of tick species was conducted through the amplification and sequencing of the following different molecular markers: 12S rRNA [25] for the identification of species belonging to the *Hyalomma* genus; 16S rRNA [26] for species belonging to the *Ixodes* genus; and a partial region of TROSPA gene [27] was sequenced to confirm the identification of *Ixodes inopinatus* specimens. *Amblyomma* and *Argas* specimens were characterized by analyses of 12S rRNA, 16S rRNA, cytochrome C oxidase subunit 1 (*COI*) gene [28], 18S rRNA, and 28S rRNA [29]. All PCR products were sent to Eurofins Genomics (https://eurofinsgenomics.eu/en/custom-dna-sequencing/ Last access: 13 June 2023) for sequencing. The obtained sequences were compiled using DS Gene v1.5 software (Accelrys Inc. 2003, San Diego, CA, USA) and analyzed using NCBI’s Basic Local Alignment Search (BLAST) (https://blast.ncbi.nlm.nih.gov/Blast.cgi, last access: 8 June 2023) for the identification of tick species. The presence of the pathogens’ DNA was first tested with specific real-time PCR assays using published methods for *Rickettsia* sp. [30], *R. aeschlimannii* [31], and a screening assay for *Coxiella burnetii* [32], using the GoTaq real-time PCR Master Mix (Promega, Madison, WI, USA) and the instrument QuantStudio™ 7 Flex Real-Time PCR System (Applied Biosystems, Waltham, MA, USA). Samples positive for *Rickettsia* sp. were subsequently tested for *R. aeschlimannii*. Species identification of samples positive for *Rickettsia* sp. and negative for *R. aeschlimannii* was performed by the partial sequence analysis of *OmpA* and *gltA* in accordance with the protocol previously described [33]. PCR products were visualized by microfluidic electrophoresis TapeStation 4200 (Agilent Technologies, Santa Clara, CA, USA), purified using GeneAll Expin™ PCR SV columns, and sequenced by Eurofins Genomics (https://eurofinsgenomics.eu/en/custom-dna-sequencing/, last access: 3 July 2023). Sequences were then analyzed using SeqScape v3.0 software and compared with the GenBank database using the BLAST software (https://blast.ncbi.nlm.nih.gov/Blast.cgi, last access: 1 July 2023). The *C. burnetii* real-time PCR IS1111 [32] positive samples were then further investigated by 16S rDNA [34] partial sequence (1185 bp) analysis using MEGA11 version 11.0.11 [35], by the amplification of the ten specific markers [36] used for multi-spacers typing (MST) and through *icd* real-time PCR [37].

## 3. Results

### 3.1. Bird Capture and Tick Collection

During the two-year sampling, 1340 ticks (555 in 2021 and 785 in 2022) were collected from 550 birds (235 in 2021 and 315 in 2022), with an overall mean of 2.4 ticks per bird. Infested birds belonged to 31 different species: 24 trans-Saharan migrants (No. individuals = 513, 93.3%) and seven intra-Palearctic ones (No. individuals = 37, 6.7%). Interestingly, ~77% of infested birds belonged to seven species of trans-Saharan birds (in descending order: wood warbler, common redstart, common whitethroat, whinchat, pied flycatcher, Icterine warbler, spotted flycatcher). Additionally, the same species resulted to be, in a different descending order (whinchat, common whitethroat, pied flycatcher, common redstart, wood warbler, Icterine warbler, spotted flycatcher), those with the highest infestation count, exceeding 74% of the total tick collection (Table 1).

### 3.2. Tick Identification and Pathogen Detection

Most of the ticks were immatures, mainly represented by nymphs (67.1%) followed by larvae (32.5%), and only a few female adults (0.4%). The species identification of larvae and nymphs required the use of different molecular targets that assigned them to 11 different species (Table 2). Considering the geographical origin of the tick species identified, 90.9% (*n* = 1218/1340) were sub-Saharan collected from trans-Saharan migratory birds and two specimens of an intra-Palearctic migrant (common kestrel, *Falco tinnunculus*). *Hyalomma rufipes* represented the widely preponderant species in the collection (89.2% overall), followed by a small number of *Amblyomma* ticks, mainly belonging to the *A. marmoreum* complex and one *A. variegatum*. Six specimens were identified as *H. truncatum*, and three larvae were soft ticks (Argasidae) belonging to the *Argas* genus (Table 2). One of these was identified as *Ar. persicus* (12S identity 100%, *COI* identity > 99%), while the other two, both collected from the same bird host in 2021, were described in a recent in-depth study [38] as belonging to a novel genotype strictly related to *Ar. africolumbae*. Finally, the remaining 9.1% of ticks (*n* = 122/1340) was represented by species commonly distributed in the Mediterranean Basin, found indiscriminately on both short-distance (No. ticks = 62) and long-distance migrants (No. ticks = 60) (Table 2).

Ticks positive in the real-time PCR screening for *Rickettsia* sp. were 53.8% (N = 721/1340). Among these, the great majority, 94.9% (N = 684/721), was identified as *R. aeschlimannii* and distributed among sub-Saharan ticks (96.20%; *H. rufipes* = 657/684, *H. truncatum* = 1/684), Mediterranean ticks (1.6%; *H. marginatum* = 11/684), and undefined species (2.2%; *Hyalomma* sp. = 14/684, Ixodes sp. =1/684). The DNA of the 37 specimens positive to *Rickettsia* sp. but negative to *R. aeschlimannii* was subjected to the amplification and sequencing of *gltA* and *OmpA* genes for species identification. Amplicons were obtained for 24 out of 37 positive ticks resulting in the identification of *R. monacensis* (*n* = 9), *R. helvetica* (*n* = 6), *R. felis* (*n* = 3), *R. africae* (*n* = 2), and two possible new species in Italy, namely, *R. tamurae* and *R. asembonensis*. However, for the latter species, the sequencing result also produced a high percentage identity with a rickettsial endosymbiont (Table 3). Only the two ticks genetically close to *Ar. africolumbae* assayed by real-time PCR targeting the IS1111 region for the presence of *C. burnetii* were positive (Ct 26.5–26.7). These results were also confirmed by the assay targeting the *icd* gene of the pathogen (Ct 31.9–32.4). On the other hand, *C. burnetii* MST markers were not amplified and the phylogenetic analysis of 16S partial sequence identified both samples as *Coxiella*-like endosymbionts (Figure S1, Supplementary Materials).

## 4. Discussion

During the study, we collected ticks from migratory birds during their journey from Africa to Europe with the aim to detect the introduction of African tick species and related bacterial agents, focusing on novel *Rickettsia* species and *C. burnetii*. Most of the ticks belonged to African species and were collected from trans-Saharan migrants, clearly reflecting the original wintering areas of their avian hosts. The bacterial pathogen detection in ticks revealed a high rate of infection by several *Rickettsia* species, while two samples positive for *C. burnetii* in the IS1111 real-time PCR were subsequently identified as non-pathogenic *Coxiella*-like endosymbionts.

Interestingly, the most parasitized bird species, referring to the number of infested individuals and the number of ticks/bird, were the same seven species, all trans-Saharan. This result suggests a fundamental role of these few species in the transport and spread of ticks, probably due to their feeding behavior, to the abundance of ticks in the African wintering grounds or in their stop-over sites, which deserves an in-depth dedicated study. Most of the tick specimens collected from migratory birds on Ventotene during the project were from the genus *Hyalomma* and, particularly, belonged to the species *H. rufipes*, the most abundant and widespread tick species in sub-Saharan Africa, also present in small areas of North Africa [39]. Our results corroborate previous surveys on ticks infesting migratory birds [3,4,5,6,7,8] and also confirm the route of *H. rufipes* introduced in different countries in Central and Northern Europe in the last decade [9,10,11,12,40]. Furthermore, among the African ticks potentially at risk of introduction in Italy, the detection of specimens of the genus *Amblyomma* was of considerable interest since it was allochthonous for the whole European continent and a vector for several pathogens. The only species detected both on migratory birds and mammalian hosts in Italy was *A. variegatum*, which was first reported in Sicily in 1971, and later in Sardinia and Corsica in 2018 [41,42]. Similarly, another species, genetically close to *A. marmoreum*, has recently emerged from our study and from other different surveys on migratory birds [6,43]. The identification of these immature ticks assigned to the *A. marmoreum* complex is still debated as either *A. marmoreum* or *A. nuttallii*, due to the morphological and genetic inconsistencies of the adult-type specimens used as references [43]. The work to resolve the discrepancies is ongoing in cooperation with international experts. Both the species, widespread in central and southern Africa and typically associated with reptiles (mainly tortoises and monitor lizards) in the adult stage [44,45], have never been detected on Italian ground.

In addition, the finding of soft ticks of the genus *Argas* (Argasidae) on migratory species deserves our attention. These ticks, often found on poultry and synanthropic birds, are rarely found on actively migrating species because of their feeding behavior that implies multiple short meals, usually involving a change in host [46]. Nevertheless, during our collection, two trans-Saharan migratory birds were found parasitized by three larvae: one belonging to the species *Ar. persicus*, at present considered almost ubiquitous, and two genetically related to the African *Ar. africolumbae*. Our finding of *Ar. persicus* on birds during the stop-over on an Italian island enriched the exiguous data available for this species in Italy, described with few localized records in the last century [47] and recently described in the cavities/nests of trans-Saharan migratory birds in Tuscany [48]. Conversely, the two larvae, found during our collection and recently described in detail as a novel *Argas* genotype close to *Ar. africolumbae* by Menegon and colleagues [38], represent the first record of this tick in Italy. These results, together with the data from previous surveys [5], clearly indicate that intercontinental movements are a possible, though not frequent, route of the dissemination of soft tick species as well. On the other hand, migratory birds can be exposed to ticks also at stop-over sites during their journey, as demonstrated by the modest, but not insignificant, amount of Mediterranean tick species collected by trans-Saharan birds.

Among the TBPs present in Europe and Africa, rickettsiae are widespread in both the continents and are represented by an increasing number of species, whose ecology biology, epidemiology, geographical distribution, and potential pathogenicity are often still poorly understood [49]. As for the species commonly found on migratory birds, all the new tick species detected were found infected by pathogenic bacteria mainly belonging to the *Rickettsia* genus. Indeed, our results show a high prevalence (53.8%) of these bacteria in ticks transported by migrating avifauna, mostly represented by *R. aeschlimannii*, belonging to the SFGR and responsible for human diseases. This pathogen, mainly transmitted by ticks of the *Hyalomma* genus, is widespread in the African and Eurasian Continents, Italy included [17,50]. Despite the presence of *R. aeschlimanni* being documented in ticks collected from several Italian regions [51], the incidence of documented human cases is probably limited to cases with severe symptoms and underestimated due to the difficulties in providing a correct diagnosis. Moreover, the intercontinental movements of infected ticks through birds could potentially favor the introduction of new genotypes whose pathogenicity results may not yet be known. In addition to the species extensively described in Italy, such as *R. monacensis*, *R. helvetica*, and *R. felis* (Table 3), we found ticks infected by allochthonous *Rickettsia* species, some of which have recently been reported outside the traditional endemic areas of sub-Saharan Africa. These include *R. africae*, the etiological agent of African tick-bite fever, already reported in Italy in ticks carried by migratory birds [4,6,7,8] and in the adults of *A. variegatum* found in Sardinia [42].

Of particular interest, however, is the detection of *R. tamurae* in a tick belonging to the *A. marmoreum* complex. This *Rickettsia* belongs to the human spotted fever group; however, pathogenicity in both animals and humans remains poorly understood. Its distribution is limited to the Asian continent and, to date, has been detected only in Japan and Korea [50]. *R. tamurae* was described for the first time in Japan in *A. testudinarium* in 1993, formally recognized as a novel species in 2006 [52], and then found often in association with the same tick species, typical of reptiles [53,54]. Thus, this represents the first detection of *R. tamurae* in Europe. In fact, although the detection of this species in Italy was reported in a recent systematic review [51], the authors of the cited study confirmed the identity of the detected bacteria, initially described as *R. monacensis*/*R. tamurae*, as *R. monacensis* [55]. It is not easy to explain how it could have come to Africa, and then to Europe via birds. However, we can assume a possible spread from Asia to Africa, not yet been documented, via the abundant migratory routes linking the two continents. The tree pipit (*Anthus trivialis*), the bird host of the *R. tamurae*-infected tick, might be a potential spreader having two main different “wintering” quarters, one in Africa and the other in India, reached by different populations spread over a broad breeding ground extending from Europe to all of central Asia [56]. Given the migratory flyways connection between the two continents traveled by hundreds of bird species [57], a possible first transmission of the pathogen in breeding areas, followed by the spread to other continents, cannot be ruled out. The limited studies on pathogen distribution in remote areas of Africa and central Asia explain this knowledge gap and emphasize the need for dedicated surveys. However, the common association of *R. tamurae* with ticks of the genus *Amblyomma*, parasites of reptiles in the adult stage, may suggest a kind of vector–pathogen specificity, further supporting our finding.

Conversely, the detection of a possible *R. asembonensis* in a soft tick (Argasidae) of the species *Ar. persicus* is yet to be confirmed. This pathogen has a wide distribution in the African continent and is typically transmitted by fleas; although, it has also been found in some ticks [58]. Nevertheless, its identification remains doubtful due to the high percentage of identity with a rickettsial endosymbiont and the lack of amplification and sequencing results from the *OmpA* gene.

Finally, we paid particular attention to the two *Argas* ticks that both produced positive results to *C. burnetii* by IS1111 real-time PCR. Since this target used for the detection of the pathogen is widespread in *Coxiella*-like endosymbionts of ticks [59], we investigated this by multiple approaches to shed light on the results obtained. The lack of amplification of *C. burnetii* MST markers and the phylogenetic relationship with *Coxiella*-like endosymbionts based on the 16S partial sequence analysis allowed us to assign the two samples to *Coxiella*-like and not *C. burnetii*. In this study, we also used *icd* to evaluate its potential use as a specific target for *C. burnetii*. Reeves and colleagues [60] reported the amplification of a 612-bp *icd* fragment in the *Coxiella*-like endosymbiont, while different results were achieved in a more recent study [37] where the *icd* target was not amplified from the panel of *Coxiella*-like infected ticks investigated. Based on our experience, a similar result can be ascribed to the low sensitivity of *icd*, a single copy target, rather than to the specificity of the test. In our study, indeed, we observed a mean shift of 5.6 cycles between the IS1111 and *icd* targets due to the different number of copies present in the genome. The *icd* is therefore unspecific as well as the IS1111 target, and thus useless for *C. burnetii* detection in ticks.

## 5. Conclusions

In this study, we provided a descriptive assessment of the potential arrival of allochthonous ticks and tick-borne bacterial agents, with a focus on *Rickettsia* species and *C. burnettii*, through migratory birds during their annual movements from Africa to Europe. The high percentage of immatures belonging to typical sub-Saharan tick species, mainly *H. rufipes*, confirmed the ability of long-distance migrants to introduce these arthropods into Europe. This occurrence, in light of the new environmental and ecological scenarios as consequences of global warming, which is rapidly changing the climate in the Mediterranean Basin, emphasized the increased risk of the introduction and spread of these vectors. In addition, the presence of as-yet undefined biological entities, such as those belonging to the *A. marmoreum* and *Ar. africolumbae* groups, and of other species not frequently encountered before, emphasized the knowledge gap concerning the diversity of African vectors, some of which are probably still unknown or not genetically characterized. Similarly, the high prevalence of bacterial pathogens in ticks, mainly belonging to the genus *Rickettsia*, pointed to a high level of circulation in the birds’ wintering areas and consequently to a high risk of the spread of emerging species or genotypes whose pathogenicity results in humans and European fauna are still unknown. Conversely, pathogenic *C. burnetii* was not identified in any of the collected ticks.

In conclusion, although the key role of migratory birds in the dispersal of ticks and tick-borne pathogens between continents is evident at present, this natural process cannot be avoided. Moreover, the potential number of pathogenic parasites carried by the several tick species is very high; therefore, the research, through multi-target or metagenomics approaches, is encouraged to explore a wider range of TBD agents. Increasing surveillance in Italy and the Mediterranean Basin might be a key early warning method to promptly identify any tick-borne zoonotic diseases that pose threats to human and animal health.

## Figures and Tables

**Table 1:** Infested bird species and respective tick species.

Common Name	Scientific Name	Migratory Strategy ^1^	N° Infested Birds	N° Ticks
Turtle dove	*Streptopelia turtur*	TS	1	2
Scops owl	*Otus scops*	TS	1	20
Common kestrel	*Falco tinnunculus*	IP	2	6
Golden oriole	*Oriolus oriolus*	TS	3	5
Woodchat shrike	*Lanius senator*	TS	4	4
Icterine warbler	*Hippolais icterina*	TS	47	103
Sedge warbler	*Acrocephalus schoenobaenus*	TS	8	52
Reed warbler	*Acrocephalus scirpaceus*	TS	7	12
Great reed warbler	*Acrocephalus arundinaceus*	TS	5	8
Bonelli’s warbler	*Phylloscopus bonellii*	TS	1	1
Wood warbler	*Phylloscopus sibilatrix*	TS	80	135
Willow warbler	*Phylloscopus trochilus*	TS	8	13
Chiff chaff	*Phylloscopus collybita*	IP	2	2
Sardinian warbler	*Sylvia melanocephala*	IP	2	2
Garden warbler	*Sylvia borin*	TS	7	38
Subalpine warbler	*Curruca cantillans*	TS	10	12
Common whitethroat	*Curruca communis*	TS	69	170
Song thrush	*Turdus philomelos*	IP	6	14
Spotted flycatcher	*Muscicapa striata*	TS	43	94
European robin	*Erithacus rubecula*	IP	22	35
Common nightingale	*Luscinia megarhynchos*	TS	12	29
Pied flycatcher	*Ficedula hypoleuca*	TS	52	160
Collared flycatcher	*Ficedula albicollis*	TS	3	12
Black redstart	*Phoenicurus ochruros*	IP	1	1
Common redstart	*Phoenicurus phoenicurus*	TS	76	159
Whinchat	*Saxicola rubetra*	TS	57	176
Northern wheatear	*Oenanthe oenanthe*	TS	4	12
Black-eared wheatear	*Oenanthe hispanica*	TS	1	9
Tree pipit	*Anthus trivialis*	TS	13	46
Yellow wagtail	*Motacilla flava*	TS	1	6
Serin	*Serinus serinus*	IP	2	2
Total			550	1340

^1^ TS = trans-Saharan; IP = intra-Palearctic.

**Table 2:** Species, geographic origin, and life stages of ticks parasitizing migratory birds.

Geographic Origin	Tick Species	Larvae	Nymphs	Adults	Total	%
Sub-Saharan	*Hyalomma rufipes*	391	804	-	1195	89.2
	*Hyalomma truncatum*	1	5	-	6	0.4
	*Amblyomma marmoreum* complex	1	12	-	13	1.0
	*Amblyomma variegatum*	-	1	-	1	0.1
	*Argas africolumbae* complex	2		-	2	0.1
	*Argas persicus*	1		-	1	0.1
Intra-Palearctic (Mediterranean)	*Ixodes ricinus*	3	9	-	12	0.9
	*Ixodes frontalis*	18	18	5	41	3.1
	*Ixodes ventalloi*	7	14	1	22	1.6
	*Ixodes inopinatus*	-	2	-	2	0.1
	*Hyalomma marginatum*	-	14	-	14	1.0
Undefined ^1^	*Hyalomma* sp.	11	19	-	30	2.2
	*Ixodes* sp.	-	1	-	1	0.1
Total		435	899	6	1340	100

^1^ Species not determined due to the lack of amplification for molecular targets.

**Table 3:** Results of molecular target sequencing for *Rickettsia* spp. identification.

Bird Species	Tick Species	Sequenced Targets							
		*OmpA*				*gltA*			
		Description	Query Cover	Perc. Id.	Ref.	Description	Query Cover	Perc. Id.	Ref.
*Sylvia borin*	*Ixodes ricinus*	*Rickettsia monacensis*	100%	99%	LN794217.1	*Rickettsia monacensis*	99%	100%	KU586332.1
*Erithacus rubecula*	*Ixodes frontalis*	*-*	-	-	-	*Rickettsia helvetica*	97%	99.2%	KU310588.
*Sylvia communis*	*Ixodes ventalloi*	*-*	-	-	-	*Rickettsia helvetica*	100%	100.%	KU310588.1
*Erithacus rubecula*	*Ixodes ventalloi*	*-*	-	-	-	*Rickettsia monacensis*	100%	99.7%	KU586332.1
*Erithacus rubecula*	*Ixodes ventalloi*	*Rickettsia* sp.	100%	95.6%	LC565679.1	*uncultured Rickettsia* sp.	99%	99.7%	KY753120.1
*Lanius senator*	*Hyalomma rufipes*	*-*	-	-	-	*Rickettsia felis*	99%	100%	MG952932.1
*Saxicola rubetra*	*Hyalomma rufipes*	*Rickettsia felis* isolate LIS 552A (*ompA*) gene	100%	100%	KY172885.1	*Rickettsia felis*	99%	100%	MG952932.1
*Luscinia megarhynchos*	*Ixodes ricinus*	*Rickettsia monacensis*	100%	99.8%	HM161773.1	*Rickettsia monacensis*	100%	100%	KU586332.1
*Sylvia communis*	*Ixodes inopinatus*	*Rickettsia monacensis*	99%	99.1%	LN794217.1	*Rickettsia monacensis*	93%	99.5%	KU586332.1
*Phoenicurus phoenicurus*	*Hyalomma rufipes*	*Rickettsia africae*	100%	100%	HQ335132.1	*Rickettsia africae*	100%	100%	HQ335126.1
*Luscinia megarhynchos*	*Ixodes ricinus*	*Rickettsia monacensis*	99%	100%	HM161773.1	*Rickettsia monacensis*	100%	100%	KU586332.1
*Saxicola rubetra*	*Amblyomma variegatum*	*-*	-	-	-	*Rickettsia africae*	100%	98.7%	MH751467.1
*Sylvia melanocephala*	*Ixodes ricinus*	*-*	-	-	-	*Rickettsia monacensis*	99%	99.7%	KU586332.1
*Turdus philomelos*	*Ixodes ricinus*	*Rickettsia monacensis*	99%	99.3%	MN853331.1	*Rickettsia monacensis*	100%	99.5%	KU586332.1
*Anthus trivialis*	*Amblyomma marmoreum* complex	*Rickettsia tamurae*	99%	97.8%	DQ103259.1	*Rickettsia tamurae*	100%	98.5%	KT753265.1
*Erithacus rubecula*	*Ixodes ventalloi*	*-*	-	-	-	*Rickettsia helvetica*	100%	99.2%	KU310588.1
*Sylvia cantillans*	*Ixodes ricinus*	*-*	-	-	-	*Rickettsia helvetica*	99%	100%	KU310588.1
*Phoenicurus phoenicurus*	*Hyalomma rufipes*	*-*	-	-	-	*-*	-	-	-
*Phylloscopus sibilatrix*	*Hyalomma rufipes*	*-*	-	-	-	*Rickettsia felis*	100%	99.7%	MN726355.1
*Phylloscopus sibilatrix*	*Hyalomma* sp.	*Rickettsia monacensis*	97%	100%	MK922659.1	*Rickettsia monacensis*	100%	100%	KU586332.1
*Ficedula hypoleuca*	*Ixodes ricinus*	*Rickettsia monacensis*	100%	99.3%	MN853331.1	*Rickettsia monacensis*	99%	100%	KU586332.1
*Sylvia borin*	*Ixodes ricinus*	*-*	-	-	-	*Rickettsia helvetica*	100%	99.7%	KU310588.1
*Sylvia communis*	*Argas persicus*	*-*	-	-	-	*Rickettsia endosymbiont of Haemaphysalis punctata*	100%	99.7%	EU303311.1
		*-*	-	-	-	*uncultured Rickettsia* sp.	100%	99.5%	MH673722.1
		*-*	-	-	-	*Rickettsia asembonensis*	100%	98.5%	KY445723.1
*Sylvia communis*	*Ixodes* sp.	*-*	-	-	-	*Rickettsia helvetica*	100%	100%	KU310588.1

## Data Availability

Data available upon request to the corresponding author.

## Supplementary Materials

The following supporting information can be downloaded at: https://www.mdpi.com/article/10.3390/microorganisms11082036/s1, Figure S1: Phylogenetic tree of *Coxiella* sp. created from 16S partial sequence evolutionary analyses [35,61,62].

## References

[1] Léger E., Vourc’h G., Vial L., Chevillon C., McCoy K.D. (2013). Changing Distributions of Ticks: Causes and Consequences. Exp. Appl. Acarol..

[2] Newton I. (2007). The Migration Ecology of Birds.

[3] Mancuso E., Toma L., Pascucci I., d’Alessio S.G., Marini V., Quaglia M., Riello S., Ferri A., Spina F., Serra L. (2022). Direct and Indirect Role of Migratory Birds in Spreading CCHFV and WNV: A Multidisciplinary Study on Three Stop-Over Islands in Italy. Pathogens.

[4] Rollins R.E., Schaper S., Kahlhofer C., Frangoulidis D., Strauß A.F.T., Cardinale M., Springer A., Strube C., Bakkes D.K., Becker N.S. (2021). Ticks (Acari: Ixodidae) on Birds Migrating to the Island of Ponza, Italy, and the Tick-Borne Pathogens They Carry. Ticks Tick-Borne Dis..

[5] Toma L., Mancuso E., d’Alessio S.G., Menegon M., Spina F., Pascucci I., Monaco F., Goffredo M., Di Luca M. (2021). Tick Species from Africa by Migratory Birds: A 3-Year Study in Italy. Exp. Appl. Acarol..

[6] Battisti E., Urach K., Hodžić A., Fusani L., Hufnagl P., Felsberger G., Ferroglio E., Duscher G.G. (2020). Zoonotic Pathogens in Ticks from Migratory Birds, Italy. Emerg. Infect. Dis..

[7] Pascucci I., Di Domenico M., Capobianco Dondona G., Di Gennaro A., Polci A., Capobianco Dondona A., Mancuso E., Cammà C., Savini G., Cecere J.G. (2019). Assessing the Role of Migratory Birds in the Introduction of Ticks and Tick-Borne Pathogens from African Countries: An Italian Experience. Ticks Tick-Borne Dis..

[8] Toma L., Mancini F., Di Luca M., Cecere J.G., Bianchi R., Khoury C., Quarchioni E., Manzia F., Rezza G., Ciervo A. (2014). Detection of Microbial Agents in Ticks Collected from Migratory Birds in Central Italy. Vector-Borne Zoonotic Dis..

[9] Lesiczka P.M., Daněk O., Modrý D., Hrazdilová K., Votýpka J., Zurek L. (2022). A New Report of Adult *Hyalomma marginatum* and *Hyalomma rufipes* in the Czech Republic. Ticks Tick-Borne Dis..

[10] Grandi G., Chitimia-Dobler L., Choklikitumnuey P., Strube C., Springer A., Albihn A., Jaenson T.G.T., Omazic A. (2020). First Records of Adult *Hyalomma marginatum* and *H. rufipes* Ticks (Acari: Ixodidae) in Sweden. Ticks Tick-Borne Dis..

[11] Chitimia-Dobler L., Schaper S., Rieß R., Bitterwolf K., Frangoulidis D., Bestehorn M., Springer A., Oehme R., Drehmann M., Lindau A. (2019). Imported *Hyalomma* Ticks in Germany in 2018. Parasites Vectors.

[12] Hansford K.M., Carter D., Gillingham E.L., Hernandez-Triana L.M., Chamberlain J., Cull B., McGinley L., Paul Phipps L., Medlock J.M. (2019). *Hyalomma rufipes* on an Untraveled Horse: Is This the First Evidence of *Hyalomma nymphs* Successfully Moulting in the United Kingdom?. Ticks Tick-Borne Dis..

[13] Camicas J.L., Hervy J.P., Adam F., Morel P.C. (1998). The Ticks of the World (Acarida, Ixodida): Nomenclature, Described Stages, Hosts, Distribution.

[14] Mofokeng L.S., Smit N.J., Cook C.A. (2022). Molecular Detection of Tick-Borne Bacteria from *Amblyomma* (Acari: Ixodidae) Ticks Collected from Reptiles in South Africa. Microorganisms.

[15] Mixson T.R., Campbell S.R., Gill J.S., Ginsberg H.S., Reichard M.V., Schulze T.L., Dasch G.A. (2006). Prevalence of *Ehrlichia*, *Borrelia*, and *Rickettsial* Agents in *Amblyomma americanum* (Acari: Ixodidae) Collected from Nine States. J. Med. Entomol..

[16] Ergönül Ö. (2023). The Lancet Infectious Diseases. Lancet Infect. Dis..

[17] Zhang Y.-Y., Sun Y.-Q., Chen J.-J., Teng A.-Y., Wang T., Li H., Hay S.I., Fang L.-Q., Yang Y., Liu W. (2023). Mapping the Global Distribution of Spotted Fever Group Rickettsiae: A Systematic Review with Modelling Analysis. Lancet Digit. Health.

[18] González J., González M.G., Valcárcel F., Sánchez M., Martín-Hernández R., Tercero J.M., Olmeda A.S. (2019). Prevalence of *Coxiella burnetii* (Legionellales: Coxiellaceae) Infection among Wildlife Species and the Tick *Hyalomma lusitanicum* (Acari: Ixodidae) in a Meso-Mediterranean Ecosystem. J. Med. Entomol..

[19] Bolaños-Rivero M., Carranza-Rodríguez C., Rodríguez N.F., Gutiérrez C., Pérez-Arellano J.-L. (2017). Detection of *Coxiella burnetii* DNA in Peridomestic and Wild Animals and Ticks in an Endemic Region (Canary Islands, Spain). Vector-Borne Zoonotic Dis..

[20] Ebani V.V., Mancianti F. (2021). Potential Role of Avian Populations in the Epidemiology of *Rickettsia* spp. and *Babesia* spp.. Vet. Sci..

[21] Fournier P.-E., Raoult D. (2020). Tick-Borne Spotted Fever Rickettsioses. Hunter’s Tropical Medicine and Emerging Infectious Diseases.

[22] Körner S., Makert G.R., Ulbert S., Pfeffer M., Mertens-Scholz K. (2021). The Prevalence of *Coxiella burnetii* in Hard Ticks in Europe and Their Role in Q Fever Transmission Revisited—A Systematic Review. Front. Vet. Sci..

[23] Manilla G. (1998). Acari Ixodida.

[24] Iori A., Di Giulio A., De Felici S., D’italia Z., Cringoli G., Iori A., Rinaldi L., Veneziano V., Genchi C. (2005). Mappe Parassitologiche: Zecche.

[25] Beati L., Keirans J.E. (2001). Analysis of The Systematic Relationships among Ticks of the Genera *Rhipicephalus* and *Boophilus* (Acari: Ixodidae) Based on Mitochondrial 12s Ribosomal DNA Gene Sequences and Morphological Characters. J. Parasitol..

[26] Black W.C., Piesman J. (1994). Phylogeny of Hard- and Soft-Tick Taxa (Acari: Ixodida) Based on Mitochondrial 16S rDNA Sequences. Proc. Natl. Acad. Sci. USA.

[27] Noureddine R., Chauvin A., Plantard O. (2011). Lack of Genetic Structure among Eurasian Populations of the Tick *Ixodes ricinus* Contrasts with Marked Divergence from North-African Populations. Int. J. Parasitol..

[28] Lv J., Wu S., Zhang Y., Chen Y., Feng C., Yuan X., Jia G., Deng J., Wang C., Wang Q. (2014). Assessment of Four DNA Fragments (COI, 16S rDNA, ITS2, 12S rDNA) for Species Identification of the Ixodida (Acari: Ixodida). Parasites Vectors.

[29] Hornok S., Kontschán J., Takács N., Chaber A.-L., Halajian A., Abichu G., Kamani J., Szekeres S., Plantard O. (2020). Molecular Phylogeny of *Amblyomma exornatum* and *Amblyomma transversale*, with Reinstatement of the Genus *Africaniella* (Acari: Ixodidae) for the Latter. Ticks Tick-Borne Dis..

[30] Kato C.Y., Chung I.H., Robinson L.K., Austin A.L., Dasch G.A., Massung R.F. (2013). Assessment of Real-Time PCR Assay for Detection of *Rickettsia* spp. and *Rickettsia rickettsii* in Banked Clinical Samples. J. Clin. Microbiol..

[31] Jiang J., You B.J., Liu E., Apte A., Yarina T.R., Myers T.E., Lee J.S., Francesconi S.C., O’Guinn M.L., Tsertsvadze N. (2012). Development of Three Quantitative Real-Time PCR Assays for the Detection of *Rickettsia raoultii*, *Rickettsia slovaca*, and *Rickettsia aeschlimannii* and Their Validation with Ticks from the Country of Georgia and the Republic of Azerbaijan. Ticks Tick-Borne Dis..

[32] Panning M., Kilwinski J., Greiner-Fischer S., Peters M., Kramme S., Frangoulidis D., Meyer H., Henning K., Drosten C. (2008). High Throughput Detection of *Coxiella burnetii* by Real-Time PCR with Internal Control System and Automated DNA Preparation. BMC Microbiol..

[33] Santibáñez S., Portillo A., Santibáñez P., Palomar A.M., Oteo J.A. (2013). Usefulness of Rickettsial PCR Assays for the Molecular Diagnosis of Human Rickettsioses. Enfermedades Infecc. Microbiol. Clín..

[34] Duron O., Jourdain E., McCoy K.D. (2014). Diversity and Global Distribution of the *Coxiella* Intracellular Bacterium in Seabird Ticks. Ticks Tick-Borne Dis..

[35] Tamura K., Stecher G., Kumar S. (2021). MEGA11: Molecular Evolutionary Genetics Analysis Version 11. Mol. Biol. Evol..

[36] Glazunova O., Roux V., Freylikman O., Sekeyova Z., Fournous G., Tyczka J., Tokarevich N., Kovacova E., Marrie T.J., Raoult D. (2005). *Coxiella burnetii* Genotyping. Emerg. Infect. Dis..

[37] Jourdain E., Duron O., Barry S., González-Acuña D., Sidi-Boumedine K. (2015). Molecular Methods Routinely Used to Detect *Coxiella burnetii* in Ticks Cross-React with *Coxiella*-like Bacteria. Infect. Ecol. Epidemiol..

[38] Menegon M., Casale F., Mancuso E., Di Luca M., Severini F., Monaco F., Toma L. (2023). Argas Ticks (Ixodida: Argasidae) on Migratory Birds from Africa: First Record of a Genotype Close to Argas Africolumbae in Italy. Ticks Tick-Borne Dis..

[39] Estrada-Peña A., Mihalca A.D., Petney T.N. (2017). Ticks of Europe and North Africa.

[40] Keve G., Csörgő T., Benke A., Huber A., Mórocz A., Németh Á., Kalocsa B., Tamás E.A., Gyurácz J., Kiss O. (2023). Ornithological and Molecular Evidence of a Reproducing *Hyalomma rufipes* Population under Continental Climate in Europe. Front. Vet. Sci..

[41] Pintore E., Olivieri E., Floriano A.M., Sassera D., Sanna N., Garippa G. (2021). First Detection of *Amblyomma variegatum* and Molecular Finding of Rickettsia Africae in Sardinia, Italy. Ticks Tick-Borne Dis..

[42] Cicculli V., De Lamballerie X., Charrel R., Falchi A. (2019). First Molecular Detection of Rickettsia Africae in a Tropical Bont Tick, *Amblyomma variegatum*, Collected in Corsica, France. Exp. Appl. Acarol..

[43] Hornok S., Cutajar B., Takács N., Galea N., Attard D., Coleiro C., Galea R., Keve G., Sándor A.D., Kontschán J. (2022). On the Way between Africa and Europe: Molecular Taxonomy of Ticks Collected from Birds in Malta. Ticks Tick-Borne Dis..

[44] Mihalca A.D. (2015). Ticks Imported to Europe with Exotic Reptiles. Vet. Parasitol..

[45] Horak I.G., McKay I.J., Heyne H., Spickett A.M. (2006). Hosts, Seasonality and Geographic Distribution of the South African Tortoise Tick, *Amblyomma marmoreum*. Onderstepoort. J. Vet. Res..

[46] Dietrich M., Gómez-Díaz E., McCoy K.D. (2011). Worldwide Distribution and Diversity of Seabird Ticks: Implications for the Ecology and Epidemiology of Tick-Borne Pathogens. Vector-Borne Zoonotic Dis..

[47] Pantaleoni R.A., Baratti M., Barraco L., Contini C., Cossu C.S., Filippelli M.T., Loru L., Romano M. (2010). *Argas* (*Persicargas*) *persicus* (Oken, 1818) (Ixodida: Argasidae) in Sicily with Considerations about Its Italian and West-Mediterranean Distribution. Parasite.

[48] Monti F., Baratti M., Viviano A., Mori E. (2023). Ticks in the Box: *Argas persicus* Occurrence in Nest Boxes of Secondary Cavity-Nesting Bird Species in Italy. Eur. J. Wildl. Res..

[49] Tomassone L., Portillo A., Nováková M., De Sousa R., Oteo J.A. (2018). Neglected aspects of tick-borne rickettsioses. Parasites Vectors.

[50] Piotrowski M., Rymaszewska A. (2020). Expansion of Tick-Borne Rickettsioses in the World. Microorganisms.

[51] Guccione C., Colomba C., Tolomeo M., Trizzino M., Iaria C., Cascio A. (2021). Rickettsiales in Italy. Pathogens.

[52] Fournier P.-E., Takada N., Fujita H., Raoult D. (2006). *Rickettsia Tamurae* sp. nov., Isolated from *Amblyomma testudinarium* Ticks. Int. J. Syst. Evol. Microbiol..

[53] Thu M.J., Qiu Y., Matsuno K., Kajihara M., Mori-Kajihara A., Omori R., Monma N., Chiba K., Seto J., Gokuden M. (2019). Diversity of Spotted Fever Group Rickettsiae and Their Association with Host Ticks in Japan. Sci. Rep..

[54] Motoi Y., Asano M., Inokuma H., Ando S., Kawabata H., Takano A., Suzuki M. (2013). Detection of *Rickettsia tamurae* DNA in Ticks and Wild Boar (*Sus scrofa leucomystax*) Skins in Shimane Prefecture, Japan. J. Vet. Med. Sci..

[55] Baráková I., Derdáková M., Selyemová D., Chvostáč M., Špitalská E., Rosso F., Collini M., Rosà R., Tagliapietra V., Girardi M. (2018). Tick-Borne Pathogens and Their Reservoir Hosts in Northern Italy. Ticks Tick-Borne Dis..

[56] BirdLife International (2023). Species Factsheet: Anthus trivialis. http://datazone.birdlife.org/species/factsheet/tree-pipit-anthus-trivialis.

[57] BirdLife International (2010). Spotlight on Flyways. Presented as Part of the BirdLife State of the World’s Birds Website.

[58] Maina A.N., Jiang J., Luce-Fedrow A., St. John H.K., Farris C.M., Richards A.L. (2019). Worldwide Presence and Features of Flea-Borne *Rickettsia asembonensis*. Front. Vet. Sci..

[59] Duron O. (2015). The IS1111 insertion sequence used for detection of *Coxiella burnetii* is widespread in *Coxiella*-like endosymbionts of ticks. FEMS Microbiol. Lett..

[60] Reeves W.K., Loftis A.D., Sanders F., Spinks M.D., Wills W., Denison A.M., Dasch G.A. (2006). *Borrelia*, *Coxiella*, and *Rickettsia* in *Carios capensis* (Acari: Argasidae) from a Brown Pelican (*Pelecanus occidentalis*) Rookery in South Carolina, USA. Exp. Appl. Acarol..

[61] Hasegawa M., Kishino H., Yano T. (1985). Dating the human-ape split by a molecular clock of mitochondrial DNA. J. Mol. Evol..

[62] Felsenstein J. (1985). Confidence limits on phylogenies: An approach using the bootstrap. Evolution.

