# Supplementary material for: Tick Species Diversity and Molecular Identification of Spotted Fever Group Rickettsiae Collected from Migratory Birds Arriving from Africa
Source: Microorganisms. 2023 Aug 8;11(8):2036. doi: 10.3390/microorganisms11082036 (PMC10458931; doi:10.3390/microorganisms11082036)
Supplement: Supplementary file 1 [file microorganisms-11-02036-s001.zip › microorganisms-2540733-supplementary.pdf]

Figure S1. Phylogenetic tree of *Coxiella* sp. resulted by 16S partial sequence evolutionary analyses.

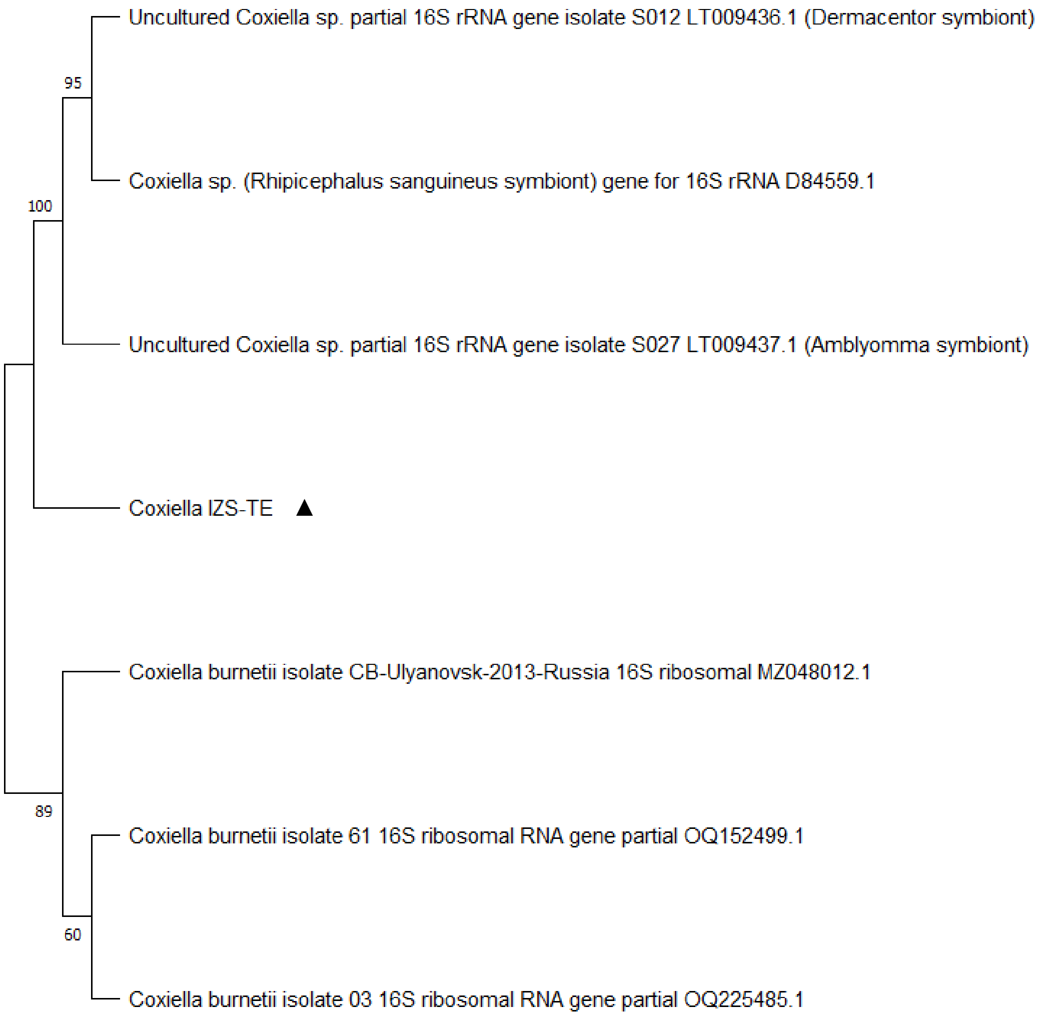

Note: The evolutionary history was inferred by using the Maximum Likelihood method and Hasegawa-Kishino-Yano model [1]. The bootstrap consensus tree inferred from 100 replicates [2] is taken to represent the evolutionary history of the taxa analyzed. Branches corresponding to partitions reproduced in less than 50% bootstrap replicates are collapsed. The percentage of replicate trees in which the associated taxa clustered together in the bootstrap test 100 replicates)are shown next to the branches [2]. Initial tree(s) for the heuristic search were obtained automatically by applying Neighbor-Join and BioNJ algorithms to a matrix of pairwise distances estimated using the Maximum Composite Likelihood (MCL) approach, and then selecting the topology with superior log likelihood value. The rate variation model allowed for some sites to be evolutionarily invariable ([+*I*], 45.61% sites). This analysis involved 7 nucleotide sequences. Codon positions included were 1st+2nd+3rd+Noncoding. There were a total of 1185 positions in the final dataset. Evolutionary analyses were conducted in MEGA11 [3]. ▲= specimens detected in this study.

1. Hasegawa, M.; Kishino, H.; Yano, T. Dating the human-ape split by a molecular clock of mitochondrial DNA. *Journal of Molecular Evolution* **1985**, **22**:160-174.
2. Felsenstein J. Confidence limits on phylogenies: An approach using the bootstrap. *Evolution* **1985**, 39:783-791.
3. Tamura, K.; Stecher, G.; and Kumar, S. MEGA 11: Molecular Evolutionary Genetics Analysis Version 11. *Molecular Biology and Evolution* **2021**, https://doi.org/10.1093/molbev/msab120.
